# RNA interference: a multifaceted innate antiviral defense

**DOI:** 10.1186/1742-4690-5-17

**Published:** 2008-02-01

**Authors:** Ajit Kumar

**Affiliations:** 1 Department of Biochemistry and Molecular Biology. George Washington University School of Medicine. 2300 I Street, N.W. Washington, D.C 20037, USA

## Abstract

The RNA interference mechanism utilizes short RNA duplexes to either suppress or induce target gene expression. Post-transcriptional regulation mediated by microRNA is an integral component of innate antiviral defense. The magnitude and the efficiency of viral restriction guided by RNA-based defenses, as well as the full physiological implication of host-pathogen engagement, constitute exciting areas of investigation in the biology of non-coding RNAs.

RNA interference (RNAi) is a highly conserved mechanism for gene silencing in higher eukaryotes [1]. RNAi-based gene silencing utilizes 21-nucleotide duplexes (short interfering RNA or siRNA) consisting of 19 base pairs with 2 nucleotides overhanging each 3' ends. These are derived from longer double-stranded RNA by sequential cleavage with the RNase III enzymes, Drosha and Dicer [2-5]. The microRNAs (miRNAs) are transcribed as mRNA-like primary miRNA (pri-miRNA) and then processed by the nuclear enzyme Drosha into shorter stem-loop structures (pre-miRNAs) that are exported from the nucleus to the cytoplasm, where they are cleaved by Dicer to yield ~21–23 nucleotide long miRNAs containing 5'-phosphate and 3'-hydroxyl termini [2]. Although the two small RNAi effecter molecules have different origins (siRNA is derived from double-stranded RNA transcripts and miRNAs are derived from longer stem-loop structures), both are incorporated into the RNA-induced silencing complex (RISC) for mRNA targeting. In practice, synthetic siRNA duplexes are widely used for loss of function analysis, while endogenous RNAi utilizes miRNAs [6].

Unlike the perfect base-complementarity of the siRNA 'guide' strand and its target mRNA, miRNAs bind their target mRNAs primarily through their 5' nucleotides 2–7, also known as the "seed" sequence. Base-pairing at the seed sequence is important for miRNA-mRNA interaction. However, perfect seed sequence pairing is not always essential for effective translational inhibition of the target mRNA by the miRNA. Thus, a single miRNA could regulate multiple mRNA targets during the stress response [7], and conversely, host miRNAs could target viral RNAs synthesized in mammalian cells in order to defend against infection [8,9].

A number of recent studies using primary cells have shown that the innate RNAi response is an important component of the mammalian antiviral response. This conclusion was reached based on several findings. Firstly, depletion of RNAi function leads to enhanced viral replication in infected cells. Because of its essential role in processing small hairpin RNA (shRNA) to generate small interfering RNA (siRNA), and its function in producing mature miRNA from pre-miRNA, depletion of Dicer has proven to be a useful tool in investigating the significance of RNAi in viral replication. Intriguingly, mice with attenuated Dicer-1 expression were recently demonstrated to be impaired in miRNA production and were more susceptible to vesicular stomatitis virus (VSV) replication [10]. This finding also provided *in vivo *genetic evidence that specific miRNAs, miR24 and miR93, can influence viral growth in mammals. As expected, mutant VSV (M2) lacking miR24 and miR93 targeting sites were more pathogenic in mice than the wild-type VSV was.

Secondly, a number of reports offer evidence for direct contributions by human miRNAs in regulating retroviral replication. Thus, human miR32 has been shown to limit the replication of primate foamy virus type 1 (PFV-1, a retrovirus akin to HIV-1) [11]. The human miR17/93 cluster was found to impact virus replication in peripheral blood mononuclear cells isolated from HIV-1 infected patients [12]. Additionally, Huang *et al. *[8] recently reported that a cluster of cellular miRNAs (miR-28, miR-125b, miR-150, miR-223 and miR-382) are enriched in resting CD4^+ ^T-cells and may be responsible for directly restricting HIV-1 expression. While the overall quantitative contribution of host cell miRNAs to combating infection still needs to be assessed (for example, individual inhibition of specific miRNAs only modestly relieved the inhibition of virus production, whereas a combination of the five miRNA inhibitors substantially increased virus production in several different HIV strains), it appears from the study by Huang *et al. *[8] that cellular miRNAs contribute to post-integration latency in HIV-1 infected individuals by recognizing target sequences in 3' UTR of viral mRNAs.

Thirdly, in another viral system, the hepatitis C virus (HCV), cellular miRNAs are apparently a component of the down stream antiviral effectors of the interferon response pathway. In a recent report, Pederson *et al. *[9] found that IFNβ treatment induced the expression of several cellular miRNAs; eight of these miRNAs have sequence-predicted targets within HCV genomic RNA. The physiological significance of IFNβ-induced miRNAs in regulating HCV replication was established in experiments which blocked these miRNAs using specific antagomirs. Treatment with the antagomirs resulted in the neutralization of IFNβ-mediated antiviral effects.

The evidence for RNA virus-derived miRNAs that influence host gene expression in order to promote viral replication is indirect. However, DNA virus-encoded miRNAs such as HSV-1 miR-LAT, or the SV40 late strand derived miRNA, constitute a direct role for viral miRNAs in modulating host-defenses. The latency-associated transcript (LAT) of herpes simplex virus-1 (HSV-1) is known to inhibit apoptosis and maintain latently infected neurons by generating micro-RNA (miR-LAT, from the exon 1 region of HSV-1 LAT gene) which downregulates the expression of transforming growth factor (TGF-β) and SMAD3 [13]. Thus miR-LAT directly contributes to HSV persistence in a latent form in sensory neurons. SV40 late strand-encoded miRNAs overlap with the viral early mRNA produced from the opposite strand, thus reducing the expression of large T antigen late in the viral replicative cycle [14]. Since SV40 large T antigen is the major target of the host cytotoxic T lymphocyte (CTL) response, its downregulation by the viral miRNA allows evasion of host immune surveillance and persistence of the viral infection.

Lastly, the significance of a host miRNA antiviral-defense is consistent with an increasing number of studies suggesting that mammalian viruses have evolved mechanisms that enable them to evade RNAi-based restrictions. Several viruses have exploited the cellular protein Dicer to process small viral RNAs in order to impact viral replication [15-17]. Additionally, discrete virus-encoded functions have been shown to subvert cellular RNAi activity [18-26]. Currently, the magnitude and efficiency of these RNAi-suppressive functions remain controversial. However, these functions are supported by new data suggesting that the status of cellular RNAi can be dramatically influenced by the physiological stress conditions of the cell [7]. In a recent report, Stern-Ginossar et al., identified major histocompatibility complex class I-related chain B (MICB) as the target of hcmv-miR-Ul122 [27]. MICB is a stress-induced ligand of the natural killer (NK) cell activating receptor NKG2D, which is critical for the NK cell killing of virus-infected cells. This intriguing finding directly links a virus-encoded miRNA to downregulation of the host immune defense.

At this stage of our understanding, it remains unclear how best to model authentic host-virus physiology when studying miRNAs. For instance, Randall *et al. *[28] recently explored the role of RNAi in regulating HCV replication. The authors employed siRNA-directed loss of function of 62 host cell genes that were known to interact with either HCV RNA or protein to gain an understanding of host-virus interactions. For the most part, depletion of the HCV-interacting genes resulted in inhibition of viral RNA replication with a parallel decline in the release of infectious virus particles. On closer examination, however, the physiological interpretation becomes less clear, since the experiments were conducted using either sub-genomic replicons or replication competent chimeric HCV genomes, but not with authentic HCV strains. The distinction holds added importance when the issue shifts to the role of RNAi/miRNA in viral infection. Here, a particularly intriguing question is the contribution of human miR-122 to the modulation of HCV replication. MiR-122 targets HCV RNA within its highly conserved 5' UTR, and miR-122 appears to be required for viral RNA replication [29]. Yet, perplexingly, there are cell types that lack miR-122 expression and still support HCV replication. Thus, the physiological necessity of miR-122 for HCV replication remains incompletely established particularly since all extant studies employed hepatoma cell lines challenged with either sub-genomic HCV replicons or a replication-competent chimeric HCV genome constructed from two different forms of HCV (genotype 2a). As yet, there are no studies that directly examine the impact of miR-122 on native HCV replication in primary hepatocytes.

Endogenous microRNAs (miRNAs) are carefully controlled cell-type specific regulatory molecules that modulate mRNA expression. Introduction of RNA duplexes into cells can increase or decrease the expression of genes [30]. For example, RNA duplexes targeted to the progesterone receptor (PR) promoter resulted in increased expression of PR RNA and protein [31]. It is yet unclear if the activating RNAs bind directly to DNA, the RNA transcript upstream of the transcription start site or to the antisense transcript. Moreover, miRNA/RNAi functions in primary cells may be quite distinct from those in tumor cell lines. Even though computer based predictions of miRNAs and their targets have seen remarkable improvements in the recent past, functional validation of miRNA-targets in specific tissues and cell types lags far behind [32]. In particular, we need a better understanding of the intracellular compartmentalization and trafficking of miRNAs to make a determination on how specific miRNAs modulate their targets under different physiological conditions [33]. The RNAi directed by endogenous cellular miRNAs represents a greater complexity when compared to that conducted by exogenous synthetic siRNAs, and the need to use primary cells and authentic viral infections puts considerable limitations in interpreting the significance of RNAi responses demonstrated from non-physiological settings (e.g. chimeric HCV replication in tumor cells).

Of caution, several studies suggest that miRNA regulation may be vastly different in tumor cells than in primary cells. In a recent analysis of miRNA expression profiles during antigen specific T cell differentiation [34,35], the authors noted dynamic changes in the expression of miRNAs. Importantly, Wu et al., [35] argue that a given miRNA hairpin may generate more than one product. Therefore, the designation of a particular sequence for a given miRNA may not adequately describe all the forms of miRNA present within a cell. Such variations might affect the stability or subcellular localization or miRNA target specificity. We are at the early stages of appreciating the influence of structural domains in miRNA that influence its functional efficacy. Moreover, cellular ribonucleoprotein (RNP) complexes may serve as a reservoir of ncRNA-based post-transcriptional gene regulatory signals [36]. A significant next challenge in miRNA studies may require that we understand better how these reservoirs, RNA-protein complexes, RNA-binding proteins and RNA-editing enzymes are choreographed (Figure 1) to counteract/guide miRNA function [37-40]. Thus, while current findings support the relevant interactions between viruses and mammalian cellular miRNAs, the full physiological implication of this host-pathogen engagement awaits further details and additional investigation.

**Figure 1:**
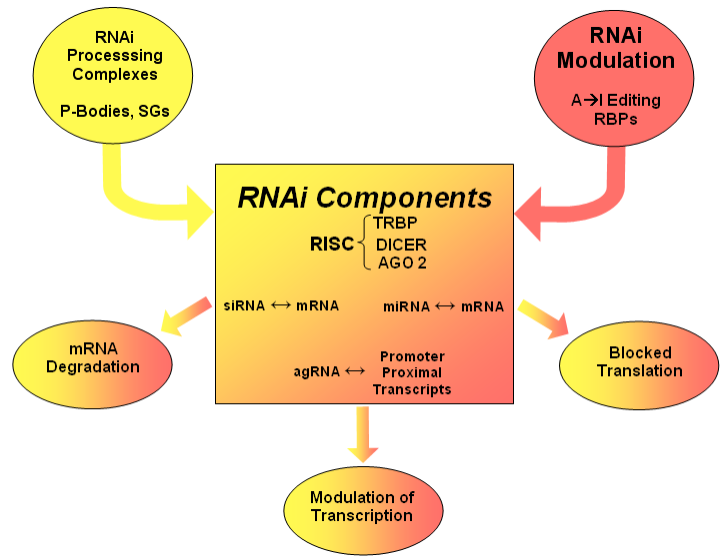
Outlines the regulatory influence of intracellular RNA-Protein complexes, such as processing bodies, P-Bodies and stress granules, SGs; the RNA-binding proteins RBPs and the A-to-I RNA-editing that influence the efficiency of target site recognition and miRNA function (36-40), and are likely to influence RNAi and antiviral defense.
